# Juvenile Protective Association

**Published:** 1908-03

**Authors:** 


### JUVENILE PROTECTIVE ASSOCIATION.

---

Recently the Juvenile Protective Association for the Southern and Central States was organized in Atlanta with the purpose of preventing wayward children, especially those being reared in an unfavorable invironment, from becoming criminals in after years.

This worthy movement should be encouraged in every possible way, for during childhood the pliable character is easily moulded for a subsequent career as a good citizen or as a criminal, and no motive could be more commendable than one with the avowed purpose of improving the chances for such children.

The objects of the Association are:

First. To erect a Juvenile State, modeled after the George Junior Republic, at Freeville, N. Y.

Second. To cause to be established Juvenile Courts and probation systems in central and southern states.

Third. To see that there is adequate legislation to carry out the plan for rescuing juvenile offenders.

This movement was started in Atlanta six years ago, and with results so encouraging that it now seems safe to predict that great and lasting good may be expected by this permanent organization.

Dr. L. G. Hardman was elected president, C. L. Anderson, first vice-president; D. S. Houseman, second vice-president; J. E. Annis, third vice-president; Crawford Jackson, general secretary; W. R. Hammond, treasurer.

It was announced that the Association had received a bond for title to a large and fine tract of land six miles from Athens and twelve miles from Commerce, Ga., from Dr. L. G. Hardman, of Commerce, who will give a guarantee deed as soon as the Association raises the needed $20,000 for the proposed improvements in establishing the Juvenile State where homeless and wayward children will be looked after.

The following trustees were elected: Judge W. R. Hammond, Dr. W. W. Landrum, General Clifford L. Anderson, Dr. Frank Eastman, Rev. Crawford Jackson, of Atlanta; J. L. Mann, of Florence, S. C., L. O. Patterson, of Greenville, S. C.; E. C. Brooks, of Goldsboro, N. C.; Rev. J. B. Lee and George W. Wilson, of Jacksonville, Fla.; D. S. Houseman, of Montgomery, Ala.; Rev. W. H. LaPrade, of Jackson, Miss.; Rev. J. J. Chisolm, of Natchez, Miss.; L. P. Brown, of Meridian, Miss.; J. E. Annis, of Chattanooga, Tenn.; Rev Preston Blake, of Lexington, Ky.; Asa G. Candler, of Atlanta; A. O. Granger, of Cartersville, Ga.; Dr. L. G. Hardman, of Commerce, Ga.; O. C. Wysong and J. S. Cox, of Greensboro, N. C.; Barnett Lewis, of Richmond, Va.; Robert Tait, of Norfolk, Va.; James L. Anderson and W. H. Witham, of Atlanta; Dr. B. J. Baldwin, of Montgomery, Ala.; E. Craighead, of Mobile, Ala.

